# Calcium, magnesium, and subarachnoid hemorrhage

**DOI:** 10.18632/aging.101543

**Published:** 2018-08-31

**Authors:** Anil Can, Rose Du

**Affiliations:** 1 Department of Neurosurgery, Brigham and Women’s Hospital, Harvard Medical School, Boston, MA 02115, USA; 2 Channing Division of Network Medicine, Brigham and Women’s Hospital, Boston, MA02115, USA

**Keywords:** hypocalcemia, hypomagnesemia, aneurysmal subarachnoid hemorrhage

Despite improvements in diagnosis and treatment of intracranial aneurysms (IA), subarachnoid hemorrhage caused by rupture of IAs remains a devastating disease. While traditional risk factors, such as aneurysm size, morphology, smoking and hypertension, partially account for intracranial aneurysm rupture risk, many patients fall into a grey-zone in whom identification of additional novel modifiable risk factors and biomarkers may be beneficial and lead to improved outcomes.

Although recent evidence suggests that hypocalcemia and hypomagnesemia are significantly associated with the extent of bleeding in patients with ICH, and the role of magnesium has been studied in the context of post-aSAH complications and outcome, the role of calcium and magnesium in the context of risk of aneurysm rupture is unknown [1–3]. Therefore, we recently investigated the magnitude and direction of the association between total serum calcium and magnesium values at admission, and the risk of aneurysmal subarachnoid hemorrhage [4]. In multivariable analysis both serum total calcium (OR 0.31, 95% CI 0.25-0.39) and serum magnesium (OR 0.60, 95% CI 0.41-0.89) at diagnosis were significantly and inversely associated with ruptured intracranial aneurysms [4]. These findings are in line with other authors who demonstrated a relationship between low admission serum calcium levels and larger hematoma volume and hematoma expansion in patients with acute ICH [1–3]. Calcium plays an important role in activation of factors IX, X (by IXa), VIIIa, phospholipid, factor X (by tissue factor and VIIA), cleavage of prothrombin to thrombin (by prothrombinase), and crosslinking of fibrin (by factor XIIIa) [5]. Thus, low levels of calcium may lead to aneurysmal subarachnoid hemorrhage by impairing platelet function and affecting the coagulation cascade. Indeed, in the subgroup of patients who were not on anticoagulation therapy, patients with hypocalcemia had significantly higher INR values than non-hypocalcemic patients, supporting calcium's role in the coagulation pathway [4]. Moreover, we recently demonstrated in another case-control study of 4,696 patients with 1,300 ruptured aneurysms, that elevated INR values are significantly associated with an increased risk of aSAH [6]. In addition to hypocalcemia, low magnesium values on admission were also significantly associated with ruptured intracranial aneurysms [4]. Indeed, it has been shown that magnesium ions significantly augment the biological activities of factor IX [7]. However, since hypomagnesemia could also be an epiphenomenon of severe, acute brain injury, the question remains whether hypomagnesemia is associated with the cause or the effect of aneurysmal subarachnoid hemorrhage [4].

Although we cannot infer causality with a biomarker association study and further prospective trials are needed to confirm these findings, the identification of biomarkers such as hypocalcemia and hypomagnesemia that are associated with rupture may provide insights into novel disease mechanisms with potential therapeutic implications.
